# The East Texas Medical and Chirurgical Society

**Published:** 1902-01

**Authors:** E. E. Guinn, W. C. Lipscomb

**Affiliations:** Secretary; Rusk, Texas; President; Rusk, Texas


### The East Texas Medical and Chirurgical Society.

Rusk, Texas, December 4, 1901.

The East Texas Medical and Chirurgical Society held its fourth semi-annual convention at Palestine, November 28 and 29, 1901.

Hon. B. S. Gardner, in an appropriate address, extended the hospitalities of the city to the visiting physicians. The president, Dr. W. C. Lipscomb, responded.

Minutes of last meeting read and approved. Twelve new members were received, towit: Drs. W. E. Davis, Elkhart; S. T. Beasley, Crockett; J. E. Howse, Austin; S. S. Barnett, Kilgore; R. R. Sample, Wiches; E. E. Bryson, Robt. Y. Lacy, Pittsburg; Jas. A. Hill, Groveton; B. F. Brown, Crockett; J. S. Collins, Crockett; J. G. Motley, Overton, and A. W. Ferguson, Montalva.

Committee on By-Laws and Constitution made a report, which was received, deferring any action until after the next State meeting.

A resolution was adopted asking the president of the State Medical Association to change next place of meeting from El Paso to some more centrally located city, as we believed this to be to the best interest of the State Medical Association.

There was also a Law and Order Committee appointed, consisting of one member from each county represented in this society, to assist the county attorney in enforcing the new medical law, the Attorney General having ruled that a permanent certificate which was issued by any district board prior to the new medical law other than at a regular meeting of said board was illegal, and if the holder of said certificate continues to practice medicine upon the authority of said certificate said holder was violating the law and is subject to the action of the courts. It being a fact that every county is infested with said illegal physicians, it is the duty of every member of this committee to inspect the physicians' register in the district clerk's office in each county and gather other evidence and present same to his county attorney. Each member of the committee is expected to present to his society at its next regular meeting a written report as to what he has done along this line. The following is the committee: Drs. W. I. M. Smith, Nacogdoches; J. M. Crawford, Alto; J. G. Motley, Overton; S. S. Barnett, Kilgore; R. C. Bristoe, Athens; T. J. Bell, Tyler; E. P. Murdock, Oakwood; J. N. Gee, Bethel; J. L. Hall, Crockett; E. E. Bryson, Pittsburg. This committee will be enlarged as the society grows. It is the duty of each member of said society to render all assistance possible to said committee.

Dr. Sam R. Burroughs was appointed as a delegate to the State society. Dr. E. B. Parsons, alternate.

Drs. E. B. Parsons, Henry Link and R. M. Dunn, of Palestine, were appointed as a Committee of Arrangements for next meeting.

Drs. J. H. Joyce, A. L. Hatchcock and S. R. Burroughs on Committee of By-Laws and Constitution.

Chairmen: General Medicine, S. N. Aston, Jewett; General Surgery, E. B. Parsons, Palestine; Obstetrics and Gynecology, Dr. E. B. Stokes, Crockett; Diseases of Children and Therapeutics, Dr. M. E. McClure, Alto.

On section work, there were several papers read and discussed, towit: "Epidemic Influenza," J. H. Paxton, Elkhart; "Typhoid Fever and its Treatment," Dr. S. N. Aston, Jewett; "Alcohol and its Use," Dr. E. E. Guinn, Rusk; "Naevi—Report of Case and Treatment by Electrolysis," Dr. R. M. Dunn, Palestine; "What to do and What Not to do in Obstetrics," Dr. T. J. Bell, Tyler; "Leucorrhea and its Treatment," Dr. R. Y. Lacy, Pittsburg; "Management of Normal Labor and After-Treatment," Dr. B. F. Chambers, Palestine; "Should Infants be Fed First Three Days of Life?" Dr. J. L. Hall, Crockett; "Symptoms and Treatment of Gonorrhea in the Female," Dr. E. P. Parsons; "Child's Second Summer," Dr. S. R. Burroughs, Buffalo.

Appropriate resolutions were adopted in behalf of Arrangements Committee, Hon. B. S. Gardner and the city.

The distinguished veteran and honorary member, Dr. R. H. Harrison, of Columbus, was present, and in a few touching remarks, tendered his thanks to the society for its action at last meeting in presenting him with a beautiful gold headed cane. While the doctor is past the three score and ten mark, still he is vigorous and is yet in active practice, and keeps abreast with the rapid march of medicine. He participated in the discussions and his remarks showed that he had not forgotten the old, old way, nor had he failed to grasp the modern way, and in some things, like many of us, he believes the old way to be the best. May the doctor's days yet be many and pleasant.

E. E. Guinn, Secretary.

W. C. Lipscomb, President.

